# 4480 Full STEM Ahead: An Innovative Approach to Translate Science into the School-community

**DOI:** 10.1017/cts.2020.269

**Published:** 2020-07-29

**Authors:** Elizabeth Hivner, William Calo, Alicia Hoke, Chelsea Keller, Jennifer Kraschnewski

**Affiliations:** 1 Penn State University; 2 Penn State College of Medicine; 3 Penn State PRO Wellness

## Abstract

OBJECTIVES/GOALS: To provide a translation of health sciences and research to a low-income population and elevate the role of science in personal health and career pathways through the implementation of a STEM-focused, researcher-led, school-community event. METHODS/STUDY POPULATION: Through a strong school district partnership, families from two urban, low-income, high-minority middle schools were invited to attend an academic-community event entitled, *Full STEM Ahead* in Lancaster, Pennsylvania. Thirty-five Penn State and community partners engaged participants in discovery-focused learning through activity stations. Topic areas included: 3D printing in medicine, herd immunity, HPV cancer prevention, lung health, and germ prevention. Evaluation data from participants and organizational partners was collected to assess process outcomes and qualitative feedback. This event was part of a randomized controlled trial to improve attitudes toward adolescent vaccination. RESULTS/ANTICIPATED RESULTS: Seventy-four parents and students participated in the two-hour event. Evaluation data indicated that 100% of participants who completed the evaluation rated the event as “good” or “excellent” and agreed that they “learned something new.” Specific qualitative feedback indicated that participants enjoyed the STEM information and various learning activities offered. School district leadership hopes to continue the partnership to host the event in future years and expand to other schools, offering an opportunity for academic-community collaboration. DISCUSSION/SIGNIFICANCE OF IMPACT: This event was an innovative approach to connect low-income communities with science and potentially effective in engaging participants in learning. Similar opportunities should be explored to bridge the gap between research and community engagement, especially to increase research awareness.

